# New tools for neuroenhancement – what about neuroethics?

**DOI:** 10.3325/cmj.2016.57.392

**Published:** 2016-08

**Authors:** Julija Erhardt, Dubravka Švob Štrac

**Affiliations:** 1 Department of Biology, Faculty of Science, University of Zagreb, Zagreb, Croatia; 2 Centre of Excellence for Integrative Bioethics, Faculty of Humanities and Social Sciences, University of Zagreb, Zagreb, Croatia; 3 Division of Molecular Medicine, Ruđer Bošković Institute, Zagreb, Croatia

## More than healthy

According to the World Health Organization (WHO), health is “... a state of complete physical, mental and social well-being and not merely the absence of disease or infirmity” (1). Today, our well-being is threatened by the ever increasing pace of life and growing demands placed on us by society. Achieving “mental and social well-being” sometimes requires mental capabilities that are beyond those that had our ancestors.

Influencing the brain in order to treat diseases and disorders is a praiseworthy and justified goal. However, enhancing the brain of healthy people who wish to be more productive and perform “better than well” and “better than their natural normal,” with the purpose to meet demands of everyday life or unhealthy ambitions, raises a multitude of ethical issues (2,3).

The absence of a clear distinction between health and disease comes to the forefront in the cases when it is not certain whether the prescription of medical treatments is justified. Should ex-patients really continue their treatments after health problems are obviously gone? What is the answer to the demands of students, academics, and business people for therapies that would provide them with increased concentration, focus, and memory?

In addition to the widely adopted and widespread pharmacological treatments for neuroenhancement, in the last decade there have emerged several non-pharmacological treatments (4,5).

## Magnetic and electric brain stimulation

A range of non-pharmacological brain treatments that in scientific and other literature are described as “non-invasive” have recently attracted a considerable interest. These methods treat the brain with a magnetic field or an electrical current and induce neuromodulatory and/or neurostimulatory effects.

The most studied and used non-pharmacological methods in both medical and non-medical environments are transcranial magnetic stimulation (TMS) and transcranial direct current stimulation (tDCS) (6,7). TMS delivers pulses of a strong magnetic field trough the scalp and induces electric current in the brain’s tissue. New TMS apparatuses are able to treat not only cortical structures, but also to reach the inner brain structures. TMS uses a sophisticated and very expensive apparatus that most often comes with an instrument allowing stereoscopic focus on the treatment area (6). However, the targeted area is still relatively large, making the specificity of this treatment questionable. In addition to the positive effects of TMS treatments in certain cases, possible side effects include local pain, headaches, and discomfort during treatments, effects on hearing, EEG after-effects, seizures, sub-clinical EEG abnormalities, syncope, cognitive/neuropsychological changes, acute psychiatric changes, endocrine after-effects, and histotoxicity. It can also have effects on various neurotransmitters, the immune system, autonomic nervous system functioning, and can stimulate adverse interactions (8).

In contrast, tDCS is so simple and affordable that there are several “tDCS do it yourself” internet sites (9,10). tDCS applies low currents to the scalp and can only modify cerebral excitability. Therefore, it is considered a neuromodulatory technique, with a more unspecific and broad treatment area compared to TMS (7). Pelletier et al (11) gave a comprehensive overview of tDCS’s side effects, including changes in neuroplasticity, neurogenesis, angiogenesis, inflammation, and apoptosis. Furthermore, tDCS treatments can affect (decrease or increase, depending on whether it is cathodal or anodal stimulation) the total number of neurite branches. It also can cause discomfort to the scalp, often cited as the only side effect.

Numerous studies indicate the usefulness of TMS and tDCS in the treatment of different brain disorders, but also their positive effects on cognition and mood (12,13). These devices are becoming widespread and their commercial use is gradually increasing, although there is no sufficient public discussion about the potential unwanted effects and consequences (14).

## Leap into the unknown

Regardless of the numerous articles about TMS and tDCS, their uncontrollable influence on brain plasticity over a considerable area of the brain tissue has not been emphasized enough. Although new approaches, which allow spatially and temporally more precise treatments, are becoming available for both techniques, they could lessen but not eliminate the harm that can be done with these treatments. In each of us, an intricate network of all our life experiences has created a complex picture of who we are. Although “only” neuromodulatory (TMS also neurostimulatory) methods, TMS and tDCS modulate not only the apparent, specific function that we want to enhance, but also various other brain functions. The serious side effects that have been described in some cases indicate that treatments with these techniques should be approached with great caution (8,11). Therefore, the tDCS and TMS outcomes and long-term effects cannot be anticipated due to their lack of specificity, individual differences, and the enormous complexity of neuronal networks. By influencing the electrical activity of the brain, ie, the firing and transfer of electrical impulses between neurons, we are changing its chemistry, neuroplasticity, and thereby various biochemical pathways crucial for the functioning of the body as a whole. This question is especially relevant and vital when these methods are applied to the developing and adolescent brain (15).

Both TMS and tDCS are applied to the surface of the scalp, and being external, these methods are perceived as mild and harmless. Their description as “non-invasive” can mask the fact that they change neural activity and therefore influence neuroplasticity. This creates unfounded trust in patients, but also in people who make their own tDCS devices for recreational use. Although capable of treating and inducing positive changes, these methods can certainly do harm as well. Hence, according to Davies and Koningsbruggen (16), the use of TMS and tDCS should comply with safety and ethical guidelines, as is the case for any surgical technique. Side effects and unintended consequences of a treatment might not only change our physiological health and hormonal balance, but also our personality and psychological profile.

In addition to safety, several other ethical issues are worth mentioning. Issues of implicit and explicit coercion in a society with growing enhancement practice are important ethical questions. Value systems, as social and cultural categories, are resistant to change. Fairness and hard work are valued highly, and an “easy approach” to achieving certain goals (exams, enrollments at prestige universities) might be perceived as inappropriate or unacceptable (17). Therefore, cognitive enhancements might share the same destiny as doping in sports. Furthermore, possible changes in users’ identity and personality, as unintended consequences of TMS and tDCS treatments, are not acceptable for most.

With the unquestionable positive effects of these techniques in certain enhancement cases, it is tempting to ask whether these treatments would be justified if they were developed to the point when they would have no side effects. However, ethical issues other than safety would still be pertinent. On the other hand, the intrinsic nature of these treatments leads us to believe that a scenario without the side effects is not possible. The acceptability of these techniques should be subject to open public discussion about their potential unwanted effects and consequences of their use.

## Instead of conclusion

Although there is still no proper public awareness of safety and ethical, social, and legal consequences of neuroenhancement, we already have tDCS devices sold at very affordable prices and “do it yourself” instructions available on the internet. TMS has also been advertised in medical institutions, promising not only treatments of brain disorders, but also mood and cognition enhancement.

Therefore, the opportunity to non-pharmacologically enhance ourselves is here before we have even had the chance to share our opinions about the regulation and control of these devices. To choose a careful and slow approach toward neuroenhancement over unhealthy ambitions and vanity might be difficult for many, because the promise of a faster progress and a better life might outweigh reasonable caution. The real question is not whether we can stop the use of these devices, but whether they are taking us in an unexpected direction with unanticipated consequences.
